# Artificial Intelligence Large Language Models in Cardiology

**DOI:** 10.31083/RCM39452

**Published:** 2025-07-08

**Authors:** Juecheng Chen, Yixiu Liang, Junbo Ge

**Affiliations:** ^1^Department of Cardiology, Zhongshan Hospital of Fudan University, Shanghai Institute of Cardiovascular Diseases, National Clinical Research Centre for Interventional Medicine, 200032 Shanghai, China; ^2^National Heart and Lung Institute, Imperial College London, Hammersmith Hospital, W120HS London, UK

## 1. What are Large Language Models?

LLMs are advanced natural language processing (NLP) systems based on deep learning (DL) techniques and trained on extensive textual datasets, enabling them to comprehend and generate human-like language. Their defining characteristics include exceptionally large parameter counts—ranging from billions to trillions and sophisticated neural network architectures, notably the Transformer, which facilitate the capture of complex linguistic relationships [1]. Increasingly, these models have been leveraged in healthcare settings for diverse applications, such as answering medical questions and automated generation of clinical documentation. With expanding institutional partnerships between developers of large language models (LLMs) and healthcare organizations, the practical implementation of these models in clinical environments is becoming progressively feasible [2]. Notable examples of general LLMs include OpenAI’s Generator Pre-Trained Transformer (GPT) series (GPT-3, GPT-4), Google’s Pathways Language Model (PaLM), Meta’s large language model meta-AI (LLaMA), and Anthropic’s Claude. Specialized medical LLMs, such as GatorTron, further emphasize their growing significance in medical applications [3].

## 2. Applications of LLMs in Cardiovascular Medicine

### 2.1 Clinical Application

The capability of LLMs to process and analyze extensive and intricate datasets offers unprecedented opportunities for enhancing diagnostic accuracy in medicine. For example, Brown *et al*. [4] utilized LLMs to incorporate social risk factors into predictive models for assessing 30-day hospital readmission following acute myocardial infarction (AMI). Dewaswala *et al*. [5] employed LLMs to analyze cardiac magnetic resonance imaging (MRI) reports to improve the detection of hypertrophic cardiomyopathy (HCM). Their models were developed for two objectives: (1) to extract HCM diagnosis and (2) to extract nine categorical concepts (such as HCM morphologic subtype, systolic anterior motion of the mitral valve) and five numeric concepts (such as maximal left ventricular (LV) wall thickness, LV mass) for phenotypic classification. As a result, their LLMs achieved an accuracy of 0.99 for extraction of HCM diagnosis from MRI reports. For numeric concepts, the accuracies were as follows: maximal LV wall thickness (0.96), LV mass (0.99), LV ejection fraction (0.98), right ventricular ejection fraction (0.99) and LV mass index (0.98) [5]. In addition, these models have demonstrated efficacy in identifying adverse drug reactions and postoperative complications [6]. By augmenting the clinicians’ abilities to interprete diagnostic results and generating comprehensive reports, LLMs facilitate clinical decision-making. A practical illustration of this is the collaborative interpretation of electrocardiograms (ECGs) by cardiologists and LLMs using conversational interfaces [7].

The potential for early identification of cardiovascular disease onset or progression through advanced analytical techniques allows for timely initiation of preventive and therapeutic interventions. Recent advancements in LLMs have enabled the processing of multimodal data, including images, thereby significantly expanding their diagnostic and predictive capabilities. The integration of ECG or cardiac MRI with clinical textual data has shown promise in enhancing risk stratification [8]. In a UK Biobank cohort, LLM-based predictive models demonstrated performance comparable to traditional risk assessment tools in forecasting major adverse cardiac events within a ten-year period [9]. Additionally, LLMs can generate evidence-based therapeutic recommendations and personalized medication regimens by referencing comprehensive drug interaction databases. Chatbot interfaces powered by LLMs have been employed to continuously monitor patient recovery parameters, such as pain levels and medication adherence, enabling dynamic adjustments to individualized care plans [10].

### 2.2 Education Application

Beyond clinical applications, LLMs are increasingly utilized in medical education. The meta-prompt functionality of LLMs enables explicit definition of conversational roles for tailored educational experiences for medical students. Modes such as ‘Socratic tutor’ encourage independent critical thinking, provide immediate responses to student inquiries, and facilitate deeper understanding of complex medical concepts [11]. The demonstrated proficiency of LLMs on cardiology and cardiac imaging board examination questions indicates their potential as personalized educational tools for clinical trainees [12]. For educators, multimodal LLMs offer streamlined methods for integrating and analyzing student-generated content across diverse formats, thereby enhancing instructional efficiency.

### 2.3 Research Application

In academic research, LLMs hold considerable promise for accelerating scientific workflows. Laboratories can employ these models to systematically extract critical insights from extensive scientific literature, discern emerging research trends, and support experimental designs [10]. LLMs are also poised to drive innovative investigations in fields traditionally considered outside the scope of textual analysis, since textual representations frequently encode information beyond human language. For instance, biological sequences and molecular structures, commonly documented through textual encodings, can be effectively analyzed using NLP methodologies embedded within LLM frameworks [13]. Additionally, the development of advanced LLMs facilitates the generation and application of synthetic data in research contexts. Detailed synthetic datasets can streamline the development and validation of clinical decision-support tools, as well as prototype automated research pipelines. By decoupling synthetic datasets from original patient records, these methods significantly mitigate privacy-related risks, thereby promoting broader accessibility and safer utilization of sensitive health information [14].

## 3. Opportunities and Challenges

LLMs exhibit transformative potential for enhancing clinical workflows through intelligent synthesis of multimodal patient data, including structured electronic health record (EHR) components and integrated imaging-text reports. This advanced cognitive capability allows contextually informed summarization of critical clinical information, effectively optimizing decision-making processes and reducing the cognitive burdens associated with clinical documentation.

LLMs also present opportunities to overcome accessibility constraints inherent to traditional healthcare systems. Streamlined, cost-effective LLMs implementations could notably benefit healthcare professionals operating within resource-limited environments, empowering them to train customized models tailored specifically to their clinical and research requirements. As computational demands decrease, it becomes increasingly feasible to develop and deploy agency-specific LLMs [2]. Moreover, the integration of LLMs with additional multimodal data sources, such as medical images, biomarkers, and wearable health, monitoring devices-enables the creation of comprehensive multimodal diagnostic and therapeutic systems, which are particularly relevant for the management of cardiovascular diseases.

Nevertheless, training LLMs requires substantial volumes of patient-derived clinical data, thereby mandating rigorous cybersecurity strategies to safeguard protected health information from unauthorized access. Implementing a robust, multi-layered security framework is essential for incorporating federated learning methodologies for decentralized data processing, role-based access controls with detailed permission structures, and blockchain-facilitated audit trails ensuring cryptographic verification throughout the data lifecycle. More specifically, patient privacy can be safeguarded through the development of anonymization tools, such as deduce, spacy, or combinations of methods [8].

Clinical deployment of LLMs necessitates vigilance concerning potential biases arising from patient demographic heterogeneity, including variations across gender, age, and race. For example, analysis of the The Cancer Genome Atlas Program (TCGA) cohort comprising 8594 tumor specimens representing 33 cancer types revealed significant ethnic disparities in sample composition: White patients constituted 82.0% of cases, followed by Black/African Americans (10.1%), Asians (7.5%), and demographically underrepresented populations (0.4%). Consequently, the vast majority of models are trained on datasets with overrepresented European ancestry populations, typically lacking systematic evaluation of algorithmic fairness [15]. Moreover, LLMs can exhibit problematic behaviors, notably generating plausible yet medically inaccurate outputs, including the fabrication of references to nonexistent scientific literature-commonly termed ‘hallucination’ [16]. For instance, hallucinations in LLMs may lead to erroneous associations between clinical, biological, or radiological features and specific diseases, potentially compromising diagnostic reliability. To mitigate such risks, developers should prioritize training models with high-quality and accurate data and conduct iterative testing to validate accuracy and quantify hallucination rates prior to clinical deployment [17].

## 4. Future Development of LLMs

The integration of multimodal architectures, which synthesize textual information with cardiovascular imaging data, outputs from biosensors or wearable devices, and genomic profiles, is poised to substantially enhance the clinical applicability of LLMs. Specifically, this integration has the potential for marked improvements in precise phenotypic characterization and the discovery of targeted therapeutic strategies.

**Fig. 1.**
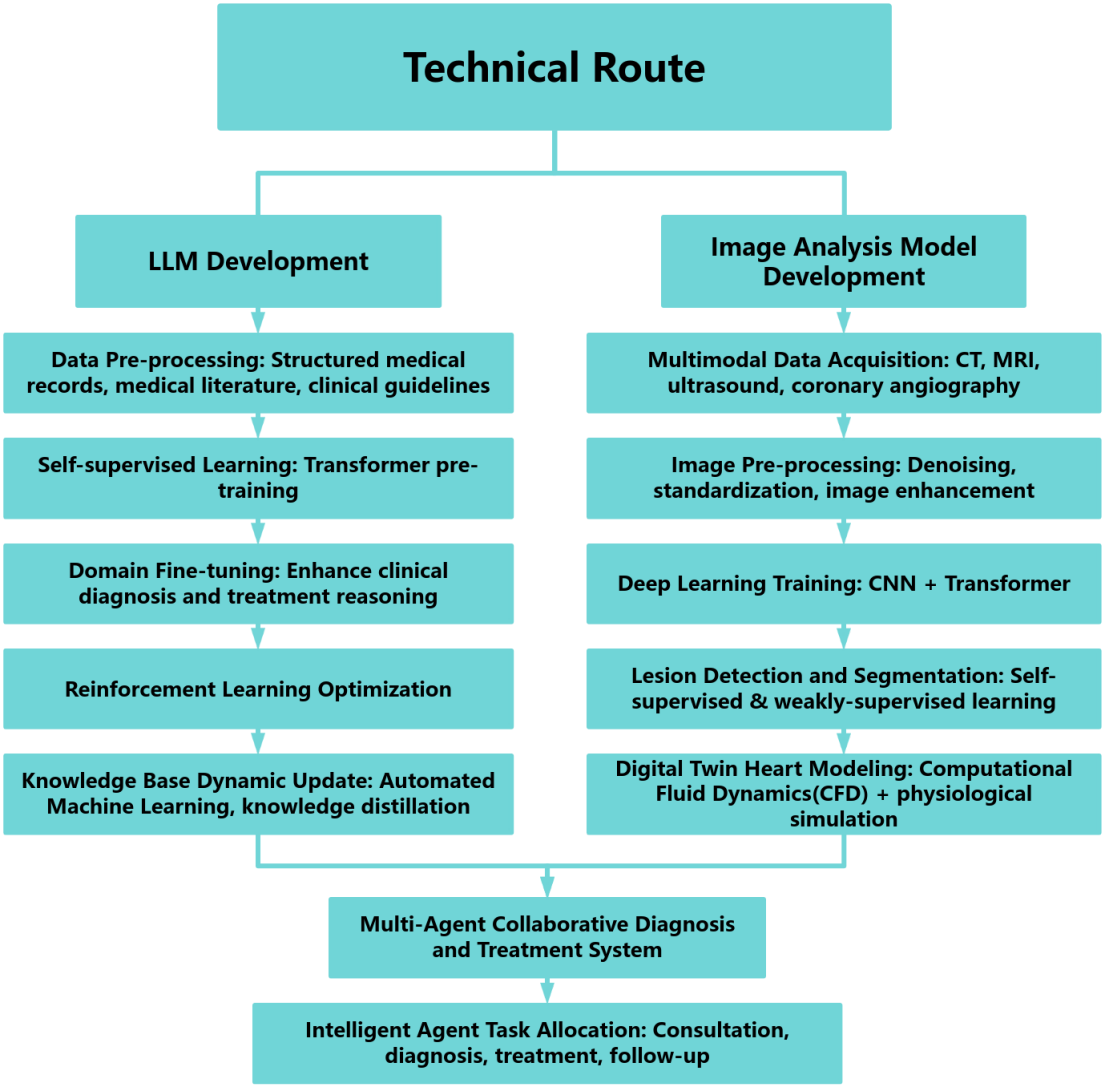
**Technical route**. LLM, large language model; CT, computed tomography; MRI, magnetic resonance imaging; CNN, Convolutional Neural Networks.

Professor Junbo Ge and his colleagues at the Zhongshan Hospital, exemplified this paradigm by developing a multimodal cardiovascular management system integrating a cardiovascular-specialized LLM pretrained on electronic health records, clinical guidelines, and biomedical literature, which was refined via domain-specific fine-tuning (Fig. 1). Complementing the LLM, their imaging model employs hybrid Convolutional Neural Networks (CNN)-Transformer architectures trained on multimodal cardiac imaging (computed tomography (CT), MRI, echocardiography, angiography) for automated lesion detection and quantitative analysis. A reinforced learning-driven multi-agent framework coordinates dynamic task allocation, cross-modal fusion, and real-time clinical decision optimization, delivering an intelligent, closed-loop cardiac care continuum from diagnosis through long-term management.

While LLMs possess transformative potential in cardiology, their integration into clinical practice necessitates a systematic approach, emphasizing iterative optimization, and rigorous validation processes [11].

## References

[1] Vaswani A, Shazeer N, Parmar N, Uszkoreit J, Jones L, Gomez AN (2017). Attention Is All You Need. *Advances in Neural Information Processing Systems*.

[2] Omiye JA, Gui H, Rezaei SJ, Zou J, Daneshjou R (2024). Large Language Models in Medicine: The Potentials and Pitfalls: A Narrative Review. *Annals of Internal Medicine*.

[3] Yang X, Chen A, PourNejatian N, Shin HC, Smith KE, Parisien C (2022). A large language model for electronic health records. *NPJ Digital Medicine*.

[4] Brown JR, Ricket IM, Reeves RM, Shah RU, Goodrich CA, Gobbel G (2022). Information Extraction From Electronic Health Records to Predict Readmission Following Acute Myocardial Infarction: Does Natural Language Processing Using Clinical Notes Improve Prediction of Readmission?. *Journal of the American Heart Association*.

[5] Dewaswala N, Chen D, Bhopalwala H, Kaggal VC, Murphy SP, Bos JM (2022). Natural language processing for identification of hypertrophic cardiomyopathy patients from cardiac magnetic resonance reports. *BMC Medical Informatics and Decision Making*.

[6] Mellia JA, Basta MN, Toyoda Y, Othman S, Elfanagely O, Morris MP (2021). Natural Language Processing in Surgery: A Systematic Review and Meta-analysis. *Annals of Surgery*.

[7] Oh J, Lee G, Bae S, Kwon JM, Choi E (2023). ECG-QA: A comprehensive question answering dataset combined with electrocardiogram. *Advances in Neural Information Processing Systems*.

[8] Boonstra MJ, Weissenbacher D, Moore JH, Gonzalez-Hernandez G, Asselbergs FW (2024). Artificial intelligence: revolutionizing cardiology with large language models. *European Heart Journal*.

[9] Han C, Kim DW, Kim S, Chan You S, Park JY, Bae S (2024). Evaluation of GPT-4 for 10-year cardiovascular risk prediction: Insights from the UK Biobank and KoGES data. *iScience*.

[10] Lee P, Bubeck S, Petro J (2023). Benefits, Limits, and Risks of GPT-4 as an AI Chatbot for Medicine. *The New England Journal of Medicine*.

[11] Gu Y, Tinn R, Cheng H, Lucas M, Usuyama N, Liu X (2022). Domain-specific language model pretraining for biomedical natural language processing. *ACM Transactions on Computing for Healthcare*.

[12] Wehbe RM (2025). Charting the future of cardiology with large language model artificial intelligence. *Nature Reviews. Cardiology*.

[13] Thirunavukarasu AJ, Ting DSJ, Elangovan K, Gutierrez L, Tan TF, Ting DSW (2023). Large language models in medicine. *Nature Medicine*.

[14] Yan C, Yan Y, Wan Z, Zhang Z, Omberg L, Guinney J (2022). A Multifaceted benchmarking of synthetic electronic health record generation models. *Nature Communications*.

[15] Chen RJ, Wang JJ, Williamson DFK, Chen TY, Lipkova J, Lu MY (2023). Algorithmic fairness in artificial intelligence for medicine and healthcare. *Nature Biomedical Engineering*.

[16] Kozaily E, Geagea M, Akdogan ER, Atkins J, Elshazly MB, Guglin M (2024). Accuracy and consistency of online large language model-based artificial intelligence chat platforms in answering patients’ questions about heart failure. *International Journal of Cardiology*.

[17] Roustan D, Bastardot F (2025). The Clinicians’ Guide to Large Language Models: A General Perspective With a Focus on Hallucinations. *Interactive Journal of Medical Research*.

